# Fast estimation of the difference between two PAM/JTT evolutionary distances in triplets of homologous sequences

**DOI:** 10.1186/1471-2105-7-529

**Published:** 2006-12-05

**Authors:** Christophe Dessimoz, Manuel Gil, Adrian Schneider, Gaston H Gonnet

**Affiliations:** 1 ETH Zurich, Institute of Computational Science, 8092 Zürich, Switzerland

## Abstract

**Background:**

The estimation of the difference between two evolutionary distances within a triplet of homologs is a common operation that is used for example to determine which of two sequences is closer to a third one. The most accurate method is currently maximum likelihood over the entire triplet. However, this approach is relatively time consuming.

**Results:**

We show that an alternative estimator, based on pairwise estimates and therefore much faster to compute, has almost the same statistical power as the maximum likelihood estimator. We also provide a numerical approximation for its variance, which could otherwise only be estimated through an expensive re-sampling approach such as bootstrapping. An extensive simulation demonstrates that the approximation delivers precise confidence intervals. To illustrate the possible applications of these results, we show how they improve the detection of asymmetric evolution, and the identification of the closest relative to a given sequence in a group of homologs.

**Conclusion:**

The results presented in this paper constitute a basis for large-scale protein cross-comparisons of pairwise evolutionary distances.

## Background

The estimation of evolutionary distances between biological sequences is at the basis of many bioinformatics problems: it plays a particularly important role in phylogenetic tree inference [1,2] and in an increasing number of comparative genomics analyses over large sets of genes or proteins (e.g. [3-5]). The most accurate way of estimating evolutionary distances is currently maximum likelihood, but the procedure is so time-consuming that is hardly practical when dealing with large datasets. In such cases, complexity is often tackled by working on the basis of individual pairs, such as in distance tree methods or in the "all-against-all" at the beginning of many comparative genomics analyses. However, by estimating an evolutionary distance for each pair individually, no knowledge about the covariance of distance estimates with common evolution can be directly obtained. Thus, when comparing pairwise distances among related sequences, for instance to infer which of two homologs is closer to a third one, confidence intervals cannot be derived directly from the pairwise estimates.

**Figure 1:**
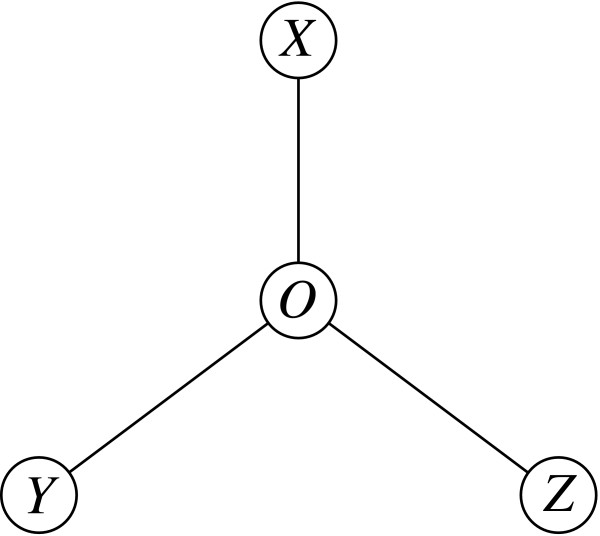
**Unrooted tree topology of all triplets of homologs**. Sequences *X*, *Y *and *Z *originating from *O*. The problem addressed here is the estimation of the difference Δ = *d*_*XY *_- *d*_*XZ *_= *d*_*OY *_- *d*_*OZ*_

The present article investigates this fundamental problem of estimating the difference between two distances in a triplet of homologs (Fig. 1). We compare the standard multivariate maximum likelihood approach with a much faster estimator based on pairwise distances, and present a formula to estimate its variance. As two examples of applications, we show how our results improve the detection of asymmetric evolution and the identification of the closest relative in a group of homologs. But first, we briefly review the Markovian model of evolution and maximum likelihood estimation of distances.

### PAM model of sequence evolution

The evolutionary distance between two biological sequences is generally based on the assumption of a first-order Markovian process of amino acid evolution. This implies two biological assumptions, common to all standard models of evolution: no memory and position-independence. The substitutional processes are described in the form of substitution matrices, defining mutation probabilities from each character to every other character for a given evolutionary distance. These matrices are either parametrical models of sequence evolution or empirically based substitution matrices. Parametrical models are often employed for nucleotide substitution (e.g. Jukes-Cantor [6] or Hasegawa-Kishino-Yano [7]), while empirical matrices (based on counted substitutions of large sets of sequence alignments) are widely used for peptide replacements in proteins. Pioneered by Dayhoff in the 1970s [8], these models have been improved with more sequence data becoming available in the 1990s (e.g. the updated Dayhoff matrices by Gonnet-Cohen-Benner [9] or Jones-Taylor-Thornton (JTT) [10]). Codon substitutions have been described by parametrical (e.g. [11]) as well as empirical (e.g. [12]) matrices.

Because of the additivity of distances computed under the Markovian model of sequence evolution. substitution matrices for a wide range of evolutionary distances can be derived from a single substitution matrix *M*(*d*_0_) through the equation *M*(*d*_0_)^*x *^= *M*(*xd*_0_), which is a special form of the Chapman-Kolmogorov equation for Markov chains. It is common and computationally more efficient to formulate this process in terms of a rate matrix *Q *from which the probability matrices for distance *d *are derived as *M*(*d*) = *e*^*dQ*^. We normally measure *d *in PAM units [8], which completely defines *Q*.

### Maximum likelihood estimation

Evolutionary distances are best estimated by maximum likelihood (ML). In case of a pair of sequences, the ML estimation is well known and practical (see *Methods *part). When more sequences are under consideration, the complexity of distance estimation by ML increases very steeply, mainly because it requires a multiple sequence alignment (MSA) and the inference of the phylogenetic tree topology, two difficult procedures for which the optimal solution can currently only be computed in exponential time with respect to the number of sequences. A common strategy for tackling this problem is to work on the basis of pairs, such as in distance tree methods. In this article, we focus on the specific problem of estimating, in a triplet of homologs *X*,*Y*,*Z *(Fig. 1). the difference Δ between two distances *d*_*XY *_and *d*_*XZ*_. In such case, the multidimensional ML approach over the triplet is still practical. We call the estimator of Δ obtained by this method $\hat{\Delta }$_*triplet*_. Alternatively, Δ can be estimated by a simple algebraic relation over pairwise distances over *X*, *Y*, *Z *estimated individually. We call this alternative estimator $\hat{\Delta }$_*pairwise*_. Details about the computation of $\hat{\Delta }$_*triplet *_and $\hat{\Delta }$_*pairwise *_are provided in the *Methods *section.

## Results and discussion

In the present section, we compare the estimators $\hat{\Delta }$_*triplet *_and $\hat{\Delta }$_*pairwise*_, and introduce a numerical approximation to estimate the variance of $\hat{\Delta }$_*pairwise*_, and show that it gives accurate confidence intervals. Finally, we describe two applications of the results.

### Comparison between the two estimators

In terms of computational complexity, the two estimators differ significantly. Given *m *sequences of length *n*, $\hat{\Delta }$_*triplet *_requires the separate treatment of each *O*(*m*^3^) triplet, and considering that an optimal 3-way alignment by dynamic programming (DP) is *O*(*n*^3^), the time complexity is *O*(*m*^3^*n*^3^). In contrast, all $\hat{\Delta }$_*pairwise *_can be computed on the basis of *O*(*m*^2^) pairs of sequences aligned by DP in *O*(*n*^2^), yielding a time complexity of *O*(*n*^2^*m*^2^). Typically, whenever an analysis involves more than a few thousand proteins, millions of triplets have to be considered and $\hat{\Delta }$_*pairwise *_is the only practical approach of the two. In terms of accuracy, both estimators are asymptotically unbiased: in the case of $\hat{\Delta }$_*triplet*_, it is a property of the ML estimator, while in the case of $\hat{\Delta }$_*pairwise*_, it is the consequence of the linearity of the expected value (see *Methods*). We compared the two estimators by simulation over a large number of triplets (length: 300 AA), generated randomly according to the PAM model of evolution with different distances *d*_*OX*_, *d*_*OY*_, *d*_*OZ *_(Fig. 1). In each experiment, both estimators were converging toward the correct value for the difference, which confirms that the asymptotic behavior is a reasonable assumption for protein sequences of typical length. In terms of statistical power; surprisingly, the observed variance of the estimates obtained by $\hat{\Delta }$_*pairwise *_was on average less than 1% larger than the observed variance of the ML estimator over the triplet, suggesting that $\hat{\Delta }$_*pairwise*_, although much faster to compute, is on average almost as accurate as $\hat{\Delta }$_*triplet*_.

The variance of $\hat{\Delta }$_*triplet *_can be computed exactly (see *Methods *section). But there is no direct estimator of the variance of $\hat{\Delta }$_*pairwise*_, since it results from an algebraic relation over pairwise distances estimated individually, whose covariances are therefore unknown. There are indirect ways of estimating that variance, through the sampling distribution when doing simulation such as the one mentioned above, or bootstrapping when handling real data. However, such procedures are very time consuming. To overcome this problem, we devised a numerical approximation of *σ*^2^($\hat{\Delta }$_*pairwise*_) as function of the pairwise distance estimates.

### Numerical approximation of *σ*^2^($\hat{\Delta }$_*pairwise*_)

In essence, the numerical approximation described here was obtained through regression over a large number of samples. We settled for this approach after discovering that the analytical solution to this problem, even when using a simpler model of evolution (all amino-acid mutations with equal probability). requires solving a polynomial of degree 23. The details of this investigation are reported in the *Appendix*. In view of this inherent complexity, the regression cannot be exact, but it turns out to be a surprisingly precise numerical approximation for comparisons that involve proteins that have an evolutionary distance smaller than 250 PAM units, which corresponds to percentage sequence identity greater or equal to 19.68%. We generated random triplets in the following way: a random-length (uniform 100..500) sequence was chosen as the origin *O*. Three random PAM distances (uniform 1..125) were selected for *d*_*OX*_, *d*_*OY *_and *d*_*OZ*_. The sequence *O *was mutated according to these distances to obtain *X*,*Y *and *Z*, our triplet. We generated about 30,000 triplets for three types of scoring matrix: updated Dayhoff matrices [9], DNA for coding genes and JTT [10]. The DNA scoring matrices were computed from a very large set of entire coding gene alignments from mammals. It is used in the OMA project [4] to align entire coding genes and is based on a 4-symbol alphabet. For each triplet, we computed pairwise distance estimates and their variances as input for the approximation. Given that $\hat{\Delta }$_*pairwise *_is almost as powerful as $\hat{\Delta }$_*triplet*_, we computed and used *σ*^2 ^($\hat{\Delta }$_*triplet*_) as reference value for *σ*^2^($\hat{\Delta }$_*pairwise*_).

We examined a large number of regressions and one approximation stood out of the rest due to its efficiency, low average error and other minor indications. Table 1 shows the coefficients of the approximation for the three types of scoring matrices.

**Table 1:** Coefficient of the approximation of *σ*^2^($\hat{\Delta }$_*pairwise*_)

Type	$\hat{d}$_*XY *_+ $\hat{d}$_*XZ*_	*σ*^2^($\hat{d}$_*XY*_) + *σ*^2^($\hat{d}$_*XZ*_)	${\hat{d}}_{YZ}^{2}$	*σ*^2^($\hat{d}$_*YZ*_)	*σ*^2^($\hat{d}$_*XY*_) *σ*^2^($\hat{d}$_*XZ*_)	error	dim
Day	-1.3090	1.0435	0.6895	-0.3339	0.1590	0.087	2.13
DNA	-1.2449	1.0933	0.6591	-0.3026	0.1181	0.098	2.13
JTT	-1.2921	1.0978	0.6741	-0.3065	0.1144	0.080	2.10

Coefficients of the regression on the logarithms for the three types of scoring matrices. The error column shows the mean error, which by virtue of being a regression on logarithms is very close to the relative error.

For example, the approximation for DNA variances is

$\begin{array}{c} {\tilde{\sigma }}^{2}({\hat{\Delta }}_{pairwise})=\frac{{\hat{d}}_{YZ}^{1.3182}}{\sigma^{2}{\left({\hat{d}}_{YZ}\right)}^{0.3026}}.\\ \frac{{\left(\sigma^{2}({\hat{d}}_{XY})+\sigma^{2}({\hat{d}}_{XZ})\right)}^{1.0933}{\left(\sigma^{2}({\hat{d}}_{XY})\sigma^{2}({\hat{d}}_{XZ})\right)}^{0.1181}}{{\left({\hat{d}}_{XY}+{\hat{d}}_{XZ}\right)}^{1.2449}} \end{array}$

Readers familiar with numerical analysis will find an analogy between the approximation presented here and standard approximations for transcendental functions. For example, it is customary to approximate *exp*(*x*) through a quotient of polynomials *p*(*x*)*/q*(*x*), for some limited range of x.

The relative error is in all the three cases less than 10%. Furthermore, since we normally use the square root of the variance, the relative error is in such cases half of the indicated. The last column indicates the dimension of the approximation which should be 2 in perfect conditions, and is indeed quite close.

The fact that very different matrices have very similar coefficients, the low error and the almost correct dimensionality reassures us of the quality of the approximation.

To test the accuracy/applicability of the approximation, as well as the other two methods to obtain the variance, we compared the 95 and 99% confidence level obtained using the appropriate number of standard deviations to the actual percentage of correct decisions obtained in a simulation over 400, 000 protein triplets generated as described above. The results are shown in Table 2.

**Table 2:** Verification of accuracy of confidence intervals

	*k *= 1.960	*k *= 2.576
|$\hat{\Delta }$_*triplet *_- Δ| > *k*·*σ*($\hat{\Delta }$_*triplet*_)	0.95129 ± 0.00067	0.99062 ± 0.00030
|$\hat{\Delta }$_*pairwise *_- Δ| > *k*·*σ*_*bootstrap*_($\hat{\Delta }$_*pairwise*_)	0.9511 ± 0.0020	0.99001 ± 0.00091
|$\hat{\Delta }$_*pairwise *_- Δ| > *k*·*σ*($\hat{\Delta }$_*triplet*_)	0.94641 ± 0.00070	0.98896 ± 0.00032
|$\hat{\Delta }$_*pairwise *_- Δ| > *k*·$\tilde{\sigma }$($\hat{\Delta }$_*pairwise*_)	0.94808 ± 0.00069	0.98953 ± 0.00032
|$\hat{\Delta }$_*pairwise *_- Δ| > *k*·*σ*_*ind*_($\hat{\Delta }$_*pairwise*_)	0.98137 ± 0.00042	0.99774 ± 0.00015

Comparison among the different methods to estimate the variance of the two estimators $\hat{\Delta }$_*triplet *_and $\hat{\Delta }$_*pairwise*_, resulting from a simulation using updated Dayhoff matrices over 400,000 proteins triplets, except for the bootstrapping method, based on 40,000 samples. The first column tests the 95% confidence interval, the second the 99% confidence interval.

As expected, the ML estimator over the entire triplet (first row) yields a precise variance estimate. On the other hand, we see that assuming independence for the estimation of the variance (last row) leads to very inaccurate confidence intervals. Estimating the variance of $\hat{\Delta }$_*pairwise *_by bootstrapping (10,000 re-samples) gives good confidence intervals, but the procedure is even more computationally intensive than $\hat{\Delta }$_*triplet*_, and therefore of little practical use in the present context. Using $\tilde{\sigma }$^2^($\hat{\Delta }$_*pairwise*_) in conjunction with the variance of the ML estimator works remarkably well (third and fourth row). And surprisingly, applying the numerical approximation (fourth row) happened to give slightly more accurate results than the exact triplet variance (third row).

**Figure 2:**
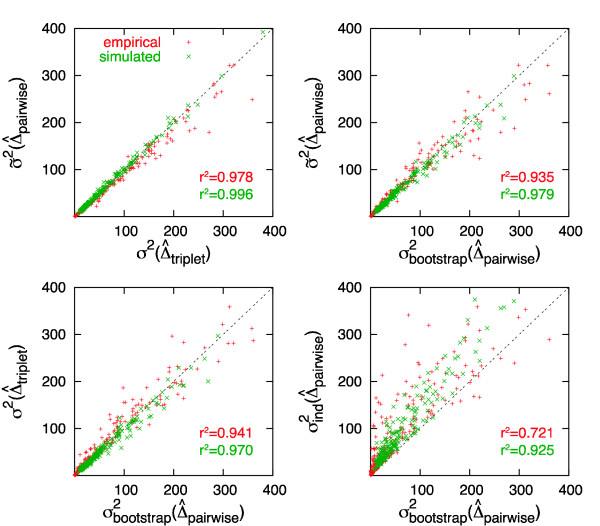
**Scatter plots comparing the variance estimators**. The upper-left plot shows the strong agreement between *σ*^2^($\hat{\Delta }$_*triplet*_) and our approximation *σ*^2^($\hat{\Delta }$_*pairwise*_). From the upper-right and the lower-left plots, it can be seen that both have similar correlation with $\sigma_{bootstrap}^{2}$($\hat{\Delta }$_*pairwise*_). Finally, the lower-right plot confirms that variance estimation under the assumption of independence can yield a large overestimation of the correct variance.

Finally, we compared the different estimators on real biological sequences, using data obtained from the OMA orthologs project [4], Triplets of orthologous sequences from various eukaryotes were randomly selected and aligned using the multiple sequence alignment package from *Darwin *[13]. All positions containing gaps were excluded, and variances were then estimated on the ungapped triplets using the various estimators (Fig. 2). The variance estimates from the approximation formula deviate very little from the results obtained by the two more expensive methods – for simulated as well as empirical alignments. Additionally, the plots illustrate the high correspondence between the results from the ML estimation and the bootstrapping, and show that the estimator based on an assumption of independence often yields overestimates of the variance. The difference between simulated and empirical data probably arises from the limitations of the Markovian model of evolution. Worth noticing is that the agreement of our estimator with bootstrapping is comparable to the one of the ML variance estimator: this implies that our approximation has a similar robustness when applied to real data.

### Applications

In the following, we provide two examples of applications that benefit from the increase in statistical power of the estimator $\hat{\Delta }$_*pairwise *_enabled by the approximation: detection of asymmetric evolution and identification of the closest relative in a set of homologs. Furthermore, in [14], we show how our result can be used in the context of paralogy detection.

We first define three indicator functions that will be used in these comparisons. They decide whether the pair of proteins *X*, *Y *is significantly closer than *X*, *Z *at the confidence level expressed by the number of standard deviations *k*. The first and second ones both use the estimator $\hat{\Delta }$_*pairwise*_, but the first definition uses as variance of the estimate the upper bound that is obtained by assuming independence of $\hat{d}$_*XY *_and $\hat{d}$_*XZ *_(see *Methods*), whereas the second use the approximation $\tilde{\sigma }$^2^($\hat{\Delta }$_*pairwise*_) of the variance. The third indicator function uses the estimator $\hat{\Delta }$_*triplet*_.

$closer_{ind}(X,Y,Z,k)=\{\begin{array}{ll} \text{true} & \text{if }{\hat{\Delta }}_{pairwise}<-k\cdot \sigma_{ind}({\hat{\Delta }}_{pairwise})\\ \text{false} & \text{otherwise} \end{array}$

$closer_{app}(X,Y,Z,k)=\{\begin{array}{ll} \text{true} & \text{if }{\hat{\Delta }}_{pairwise}<-k\cdot \tilde{\sigma }({\hat{\Delta }}_{pairwise})\\ \text{false} & \text{otherwise} \end{array}$

$closer_{triplet}(X,Y,Z,k)=\{\begin{array}{ll} \text{true} & \text{if }{\hat{\Delta }}_{triplet}<-k\cdot \sigma ({\hat{\Delta }}_{triplet})\\ \text{false} & \text{otherwise} \end{array}$

#### Asymmetric evolution

After a gene duplication, the two copies can evolve independently. It has been suggested that in many cases, one duplicate maintains the ancestral function while the other is free to evolve and acquire novel functionality [15]. This scenario implies that the protein with conserved functionality will undergo less sequence evolution than the one exploring new functionalities.

Detecting this asymmetric evolution after duplication is an important factor not only for function prediction or orthologs assignment, but also for bringing new insights in our understanding of genome evolution in general (e.g. [16-19]).

In order to identify cases of asymmetric evolution, one typically considers three sequences – the two duplicates (*Y *and *Z*)and an out-group (*X*). Several methods have been developed to test the significance of the unequal lengths of the branches leading from the common ancestor to the two duplicated sequences. Tests on simulated and real data from *Arabidopsis thaliana *for two of such methods have suggested very low statistical power to detect asymmetric evolution of duplicates [20].

The *closer *indicator function can be used to detect asymmetric evolution. With *d*_*XY *_being the distance from the out-group to the closer of the two duplicates and *d*_*XZ *_the distance to the other one, *closer *(*X*, *Y*, *Z*, *k*) decides if the two duplicated proteins have evolved at significantly different rates. The parameter *k *can be chosen to reflect the confidence level, e.g. 1.96 for the 95% level.

We tested the method using all three variants of *closer *(*k *= 1.96) on a protein set from a recent publication about whole genome duplication in *S. cerevisiae *[21]. From a set of 450 genes pairs that arose by whole genome duplication, they report 115 cases of one paralog evolving at least 50% faster than the other paralog. The position of the ancestral gene was determined by an out-group gene from *K. waltii*. Additionally, a set of 76 gene pairs is given where at least one of the *S. cerevisiae *genes evolved at least 50% faster than the *K. waltii *homolog.

**Figure 3:**
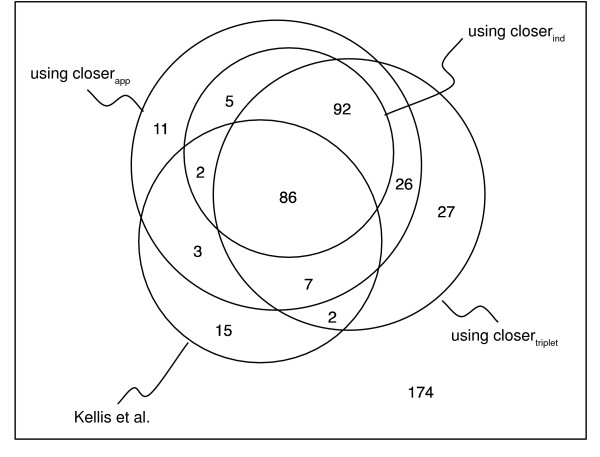
**Detection of asymmetric evolution**. Detection of Asymmetric Evolution. Comparison between the results of Kellis et al. and the three variants of *closer*, with *k *= 1.96. The circles separate cases of significant asymmetry (inside) from insignificant asymmetry (outside). For instance, there were 92 cases where all three variants of *closer *reported significant asymmetry, while the method of Kellis et al. did not detect significant asymmetry.

The results are summarized in Fig. 3. We first discuss the differences among three variants of *closer*. As expected, the over estimation of the variance of the estimator in *closer*_*ind *_considerably reduces the cases of asymmetry detected in comparison with *closer*_*app*_. As for *closer*_*app*_and *closer*_*triplet*_, they agree on 400 of 450 cases, with 21 cases only reported by *closer *_*app *_and 29 only by *closer *_*triplet*_. This discrepancy results from the error introduced by the approximation for the estimation of the variance of $\hat{\Delta }$_*pairwise*_, but mostly from the inherent differences in the predictions of the two estimators $\hat{\Delta }$_*pairwise *_and $\hat{\Delta }$_*triplet*_.

If we now compare the predictions of Kellis and colleagues with our results, it appears that in 98 out of 115 cases, their prediction of asymmetric evolution could be confirmed by *closer*_*app*_, while with the remaining 17 pairs, our method did not support the asymmetry prediction. It is remarkable, however, that all these 17 pairs belong to the group of the 76 pairs with a fast evolving *K. waltii *homolog. It seems likely that the uncertainty in placing the origin of the triplet (arising from a longer branch to the out-group) causes rate-based methods as used in [21] to report asymmetric divergence despite the unclear situation. As opposed to that, the distance-based methods presented here, by incorporating the variance of the estimates explicitly, take the uncertainty about the point of origin into account, and therefore give more conservative predictions in these cases.

Furthermore, *closer*_*app *_found 134 additional cases of asymmetry among the remaining 335 gene pairs in the data set. Together with the 98 cases above, this results in 51.6% of all genes arising from the genome duplication event. This is clearly more than the 5% that could be expected from random chance and agrees with previous studies were significant amounts of asymmetrically evolving duplicates have been reported (e.g. [22,23]).

#### Closest homolog without phylogenetic reconstruction

The identification of the closest relative of a protein (or gene) in a set of homologs traditionally requires the reconstruction of the corresponding phylogenetic tree. However, building gene trees remains a time consuming and error-prone task, thus methods based on pairwise evolutionary distance estimates are attractive. In this section, we show that using the variance approximation presented above can boost the statistical power of PAM distance comparisons to determine the closest homolog.

In simple contexts, or when accuracy is not a concern, the problem of identifying the closest relative can be solved reasonably well by coarse approaches, such as the top blast hit, or even the sequence with highest percentage identity. As the number of proteins grows larger and the number of homologs with similar distances increase, these methods show their limits. Indeed, it has been previously shown that the top blast hit is often not the closest relative [24]. At least two ideas lead to better results: the use of evolutionary distance estimates such as PAM distances, and accounting for confidence intervals, so that whenever there is not enough information to reliably discriminate among several distances, all of them are kept, presumably for further analysis.

**Figure 4:**
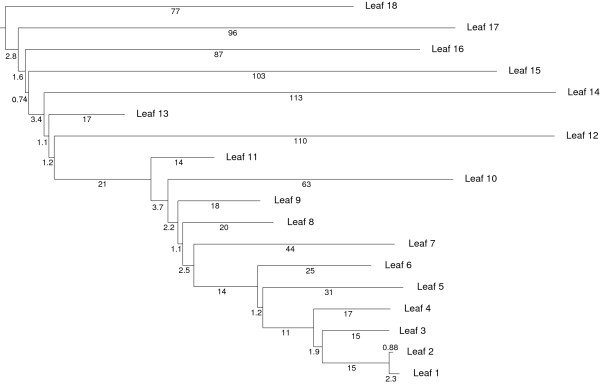
**Tree randomly generated for closest homolog simulation**. Example of a random tree (see text for description of the procedure) used to compare the different methods to infer the closest homolog to each leaf. Distances indicated are in PAM units.

Since the comparison of the methods requires precise and unbiased knowledge of the closest homolog, we use simulated data generated in the same way as in the section above, according to the PAM model. Families of homologs were created through mutation and duplication following random phylogenetic trees (Fig. 4) with the following properties: (i) each branch has a random mutation rate from a uniform distribution between 0 and 1, (ii) duplication occurs only along the leftmost branch, at random intervals, on average about every 6 PAM units, (iii) the generation is performed in 60 steps and results in trees with an average number of leaves of 13.04 (*σ *= 3.1). The very asymmetric duplication process is used to explore efficiently the parameter space, both in terms of distance magnitude to the closest homolog as in the number of homologs with similar distances.

For each protein *X *belonging to such a family, the closest homolog predictions using the following three criteria were compared to the actual closest homolog. The first one computes the subset of homologous sequences *H *that align with *X *with score higher than a particular fraction of the top score.

$Set_{1}=\{Y\in H|Score(X,Y)\geq (1-k_{1})\cdot \underset{Z\in H}{\max}(Score(X,Z))\}$

The second method computes the set of closest homologs, without using our variance approximation, formally

*Set*_2 _= {*Y *∈ *H *| ∄ *Z *∈ *H*, *Z *≠ *Y*, *closer*_*ind*_(*X*, *Z*, *Y*, *k*_2_)}

The third method computes the set of closest homologs using our approximation, formally

*Set*_3 _= {*Y *∈ *H *| ∄ *Y *∈ *H*, *Z *≠ *Y*, *closer*_*app*_(*X*, *Z*, *Y*, *k*_3_)}

The cut-off parameters *k*_1_, *k*_2_, *k*_3 _can be set according to the desired level of confidence. At *k *= 0, only the top score, respectively the shortest expected distance, is returned. Higher *k *values correspond to more conservative predictions, with increasing number of closest homolog candidates. For the evaluation of the methods, we vary *k*_1 _between 0 and 0.25, while *k*_2_, *k*_3 _are varied between 0 and 3. Note that only *k*_3 _corresponds to the number of standard deviations from the expected value.

**Figure 5:**
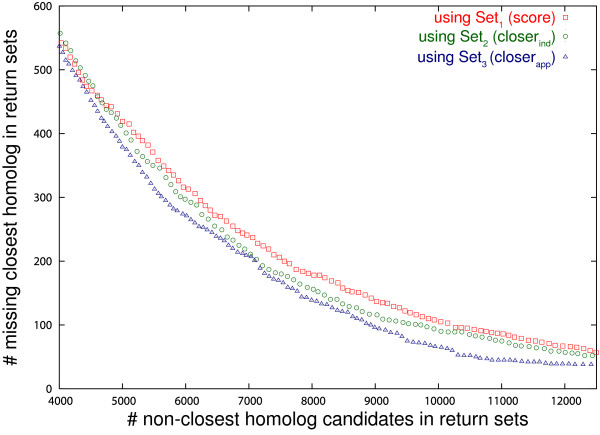
**Identification of the closest homolog**. Identification of the closest homolog: comparison between methods using alignment score (1), distance with assumption of independence (2) and distance using our variance approximation (3), on simulated data.

The resulting curves are presented in Fig. 5. At low cut-off values, all three methods perform similarly, but as *k *increases, the method using *closer*_*app *_gives better results.

## Conclusion

Computing the difference of two evolutionary distances that are not independent is a common operation in an increasing number of bioinformatics analyses. We presented and compared two estimators for the difference of two evolutionary distances in a triplet of homologs, one estimator based on pairwise distance estimates and the maximum likelihood estimator. Surprisingly, the estimator based on pairwise distance is almost as powerful as the ML estimator. But in terms of time complexity, it scales much better than the ML estimator and is therefore better suited at large-scale analyses. However, since its variance is not easy to estimate, we introduced a numerical approximation that allows the computation of accurate confidence intervals. Finally, we showed how these results can be used to test for asymmetrical evolution, and to identify the closest relative of a sequence in a group of homologs without phylogenetic reconstruction. As of future work, we plan to extend these results to models of evolution allowing rate variations, as well as insertion-deletions.

## Methods

### PAM distance estimator for a pair

The likelihood of an alignment *A *at an evolutionary distance *d *is defined [25-27] as

$\begin{array}{ccc} L(A|d) & = & \prod_{[x,y]\in A}f(x)M_{x,y}(d)\\ & = & \prod_{[x,y]\in A}f(x){\left[e^{dQ}\right]}_{x,y} \end{array}$

with *x *and *y *being aligned characters (e.g. amino acids, bases, but no deletions), and *f*(*x*) the background frequency of the character *x*. Maximizing *L*(*A *| *d*) in terms of *d *gives the ML estimator $\hat{d}$ of the evolutionary distance. This is usually done numerically using the Newton-Raphson method. The variance of the ML estimator $\hat{d}$ can be computed from the second derivative of the log-likelihood:

$\sigma^{2}(\hat{d})=-{\left(\frac{\partial^{2}L(A|\hat{d})}{\partial d^{2}}\right)}^{-1}$

Notice that the variance is obtained for free as it is already computed in Newton's iteration.

### PAM distance estimator for a triplet

#### Estimator based on pairwise distances

One can estimate Δ by performing pairwise alignments between *X *and *Y*, and between *X *and *Z*. The ML method for pairs of homologs, which was described above, computes the estimates $\hat{d}$_*XY *_and $\hat{d}$_*XZ*_. By subtracting the first from the second, an estimator for the difference is obtained:

$\hat{\Delta }$_*pairwise *_= $\hat{d}$_*XY *_- $\hat{d}$_*XZ*_

Since the two pairwise distance estimators are asymptotically unbiased and normally distributed, and considering the linearity of the expected value and the fact that the difference of two normally distributed variables is also normally distributed, the pairwise estimator $\hat{\Delta }$_*pairwise *_is also asymptotically unbiased and normally distributed, with variance

$\sigma^{2}({\hat{\Delta }}_{pairwise})=\sigma^{2}({\hat{d}}_{XY})+\sigma^{2}({\hat{d}}_{XZ})-2cov({\hat{d}}_{XY},{\hat{d}}_{XZ})$

As described above, we obtain *σ*^2^($\hat{d}$_*XY*_) and *σ*^2^($\hat{d}$_*XZ*_) from the ML distance estimation, but the process does not say anything about their covariance. If the two distances are independent, which is only the case if *d*_*OX *_= 0, the covariance is zero and the variance $\sigma_{ind}^{2}$($\hat{\Delta }$_*pairwise*_) = *σ*^2^($\hat{d}$_*XY*_) + *σ*^2^($\hat{d}$_*XZ*_) can be computed. In all other cases, $\hat{d}$_*XY *_and $\hat{d}$_*XZ *_covary and the variance of their difference is smaller than the sum of their variances. Therefore, we only have an upper bound for the variance of our estimator:

$\sigma^{2}({\hat{\Delta }}_{pairwise})\leq \sigma_{ind}^{2}({\hat{\Delta }}_{pairwise})$

Note that previous work on covariance estimation (e.g. [7,28]) do not apply here, because they require 3-way sequence alignments and are constrained to parametric models of evolution such as Jukes-Cantor and its generalizations.

#### Estimator based on triplet

Alternatively, we can estimate Δ by subtracting estimates of the distances *d*_*OY *_and *d*_*OZ*_

$\hat{\Delta }$_*triplet *_= $\hat{d}$_*OY *_- $\hat{d}$_*OZ*_

The estimates $\hat{d}$_*OY *_and $\hat{d}$_*OZ *_can be obtained by maximum likelihood over the multiple sequence alignment of *X*, *Y*, *Z *[25], in a manner analogous to the ML estimation for a pair. The likelihood *L *of a multiple sequence alignment (MSA) is the product, over all positions of the MSA, of the probability of observing characters *x*, *y*, *z *at distance *d*_*OX*_, *d*_*OY*_, *d*_*OZ *_of the origin, where such a probability is obtained by marginalizing over every character *o *at the origin:

$L(MSA|d_{OX},d_{OY},d_{OZ})=\prod_{\left[x,y,z\right]}\sum_{o\in C}f(o){\left[e^{d_{OX}Q}\right]}_{o,x}{\left[e^{d_{OY}Q}\right]}_{o,y}{\left[e^{d_{OZ}Q}\right]}_{o,z}$

where *C *is the set of characters – the 20 amino-acids in the present case, and *f*(*o*) the background frequency of the character *o*. Consequently, the log-likelihood function *l *is

$l(MSA|d_{OX},d_{OY},d_{OZ})=\sum_{\left[x,y,z\right]}\log\sum_{o\in C}f(o){\left[e^{d_{OX}Q}\right]}_{o,x}{\left[e^{d_{OY}Q}\right]}_{o,y}{\left[e^{d_{OZ}Q}\right]}_{o,z}$

The log-likelihood is maximum where its gradient disappears:

$\nabla l=\left(\begin{array}{c} \partial l/\partial d_{OX}\\ \partial l/\partial d_{OY}\\ \partial l/\partial d_{OZ} \end{array}\right)=\left(\begin{array}{c} 0\\ 0\\ 0 \end{array}\right)$

There again, the problem can be solved efficiently by Newton's iteration

${\left(\begin{array}{c} {\hat{d}}_{OX}\\ {\hat{d}}_{OY}\\ {\hat{d}}_{OZ} \end{array}\right)}_{i+1}={\left(\begin{array}{c} {\hat{d}}_{OX}\\ {\hat{d}}_{OY}\\ {\hat{d}}_{OZ} \end{array}\right)}_{i}-{\left(\nabla^{2}l_{i}\right)}^{-1}\nabla l_{i}$

where (∇^2^*l*)^-1 ^is the inverse of the Hessian (derivable in the same fashion as the gradient, not shown here). The inverse of the Hessian also yields the variance-covariance matrix of the estimates $\hat{d}$_*OX*_, $\hat{d}$_*OY*_, $\hat{d}$_*OZ *_when multiplied by -1. A final use of the Hessian is to check that its complement is positive definite, a condition necessary to ensure that the solution found is indeed a maximum and not a minimum or a saddle point. Therefore, we obtain the variance of $\hat{\Delta }$_*triplet *_from the variance-covariance matrix:

$\begin{array}{c} \sigma^{2}({\hat{\Delta }}_{triplet})=\sigma^{2}({\hat{d}}_{OY})+\sigma^{2}({\hat{d}}_{OZ})-2cov({\hat{d}}_{OY},{\hat{d}}_{OZ})\\ =[0,1,-1]{\left(-\nabla^{2}l\right)}^{-1}\left[\begin{array}{c} 0\\ 1\\ -1 \end{array}\right] \end{array}$

## Authors' contributions

CD was the main investigator and writer. MG contributed ideas, wrote part of the method section, and performed simulations. AS contributed the introduction to PAM distances, and the section on asymmetrical evolution. GG devised the numerical approximation and contributed the appendix. All authors read and approved the final manuscript.

## Appendix

### Complexity of the analytical solution of *k*-states model for triplets

In the following, we show that the analytical solution of the maximum-likelihood estimator for the distances of a triplet is very complex, even for a simplified model of mutation. The *k*-state model [29] is an idealized situation where each position has *k *possible states and the transition probabilities are all identical and only depend on the time *t*. For *k *= 4 this is equivalent to the Jukes-Cantor model [6]. Whatever is the initial state, the probability of a mutation after time *t *is given by

$p(t)=\frac{k-1}{k}(1-r^{t})$

where *r *is

$r=1-\frac{k}{100(k-1)}$

so that *t *is measured in PAM units. (Measuring in PAM units is proportional to any other measure, and it means that at *t *= 1 one percent of the characters are changed, i.e. *p*(1) = 1/100.) and that all transitions are equally likely, and only depend on the PAM distance. Under this model, the log-likelihood can be expressed in terms of the counts of matches/mismatches of the triplet (*X*, *Y*, *Z*), i.e. *N*_*xxx *_is the number of positions where all the characters are identical, *N*_*xxz *_is the number of positions where *X *and *Y *coincide but *Z *differs, etc.

$\begin{array}{c} l(A|t)=N_{xxx}\log(P_{xxx})+N_{xxz}\log(P_{xxz})+N_{xyx}\log(P_{xyx})+\\ N_{xyy}\log(P_{xyy})+N_{xyz}\log(P_{xyz})\\ P_{xxx}=(1-p_{x})(1-p_{y})(1-p_{z})+\frac{p_{x}p_{y}p_{z}}{{\left(k-1\right)}^{2}}\\ P_{xxz}=(1-p_{x})(1-p_{y})p_{z}+\frac{p_{x}p_{y}(1-p_{z})}{k-1}+\frac{(k-2)p_{x}p_{y}p_{z}}{{\left(k-1\right)}^{2}}\\ P_{xyx}=(1-p_{x})p_{y}(1-p_{z})+\frac{p_{x}(1-p_{y})p_{z}}{k-1}+\frac{(k-2)p_{x}p_{y}p_{z}}{{\left(k-1\right)}^{2}}\\ P_{xyy}=p_{x}(1-p_{y})(1-p_{z})+\frac{(1-p_{x})p_{y}p_{z}}{k-1}+\frac{(k-2)p_{x}p_{y}p_{z}}{{\left(k-1\right)}^{2}}\\ P_{xyz}=\frac{k-2}{k-1}\left((1-p_{x})p_{y}p_{z}+p_{x}(1-p_{y})p_{z}+p_{x}p_{y}(1-p_{z})+\frac{(k-3)p_{x}p_{y}p_{z}}{k-1}\right) \end{array}$

where *p*_*x *_is the probability of mutating from the origin to *X *and similarly for *p*_*y *_and *p*_*z*_. Taking partial derivatives of the likelihood with respect to *p*_*x*_, *p*_*y *_and *p*_*z *_gives a system of 3 rational polynomial equations (all the logarithms disappear) in 3 unknowns and 6 parameters. Such a system of equations has a solution that will be an algebraic function of the parameters (a root of a polynomial, where the coefficients of the polynomial involve the parameters). Despite its simple appearance, this system of equations is beyond the capabilities of current computer algebra systems to resolve. And this is not a complete surprise, as the algebraic numbers/functions involved are at least of degree 23. The special case where two of the branches have the same length, has been solved exactly in [30], they find that their solution is an algebraic function of degree 11. This unfortunately is not applicable as we are interested in the cases where the branches away from the origin are of different lengths.

We have computed the exact solution for concrete values of the parameters, in particular *N*_*xxx *_= 10, *N*_*xxz *_= 5, *N*_*xyx *_= 4, *N*_*xyy *_= 3, *N*_*xyz *_= 2, *k *= 3 using Maple and the value of *p*_*x *_is a root of the irreducible polynomial

-6582435840000 + 189590785228800 *z *- 2438333515038720 *z*^2 ^+ ...

... + 10304020514917800 *z*^21 ^- 1635488137841976 *z*^22 ^+ 99990709180560 *z*^23^

This means that the general solution will be an algebraic function of degree 23 or higher, it cannot be lower. If an instantiation of the polynomial with values gives this irreducible polynomial, then the general polynomial must be irreducible of degree 23 or higher (some terms could have simplified in the instantiation). This makes the usefulness of an exact solution inexistent. it is more difficult to solve the polynomial and select the right root than to maximize the likelihood and/or solve the system of equations by numerical methods.
